# The Mechanical Resistance of Asphalt Mixture with Steel Slag to Deformation and Skid Degradation Based on Laboratory Accelerated Heavy Loading Test

**DOI:** 10.3390/ma15030911

**Published:** 2022-01-25

**Authors:** Jiasheng Li, Jianying Yu, Shaopeng Wu, Jun Xie

**Affiliations:** State Key Laboratory of Silicate Materials of Architectures, Wuhan University of Technology, Wuhan 430070, China; lijiasheng@whut.edu.cn (J.L.); jyyu@whut.edu.cn (J.Y.); xiejun3970@whut.edu.cn (J.X.)

**Keywords:** asphalt mixture, steel slag, heavy loading, aggregate analysis, permanent deformation, skid resistance, aggregate sieving

## Abstract

Steel slag is a main form of solid waste. Using this component to replace part of the aggregate in an asphalt mixture is an effectively way of treating solid waste. To study the performance degradation of asphalt mixture with various content of steel slag under heavy loading, some large-sized basalt hot mixed asphalt mixture (BHMA) and steel slag hot mixed asphalt mixture (SHMA) in a form of specimens were prepared and a heavy loading wheel tracking test was conducted. The aggregate characteristics of basalt and steel slag were measured. The deformation and skid resistance of the asphalt mixture with different content of steel slag was tested and analyzed. Due to the particle characteristics of steel slag aggregate, it was found that a low content of steel slag can effectively improve the resistance to deformation and skid resistance of the asphalt mixture under heavy loading. The response of SHMA can still meet the pavement technical requirement after long-term heavy loading service. The main change in the mixture under heavy loading is the crushing of the 9.5–16 mm aggregate, and the content of this part also significantly affects the deformation. This study explains the mechanism of degradation of SHMA under heavy loading: the large aggregate is crushed and forms a new aggregate skeleton structure.

## 1. Introduction

In recent years, the ever-developing transportation and construction industry has required a massive quantity of aggregates. However, the limited quantity of natural aggregate resources has made it increasingly difficult to meet the demand. Therefore, it is an effective method to substitute natural aggregates with recycled aggregates and other resources [1,2,3,4].

As an industrial by-product generated from steel tailing waste, with the development of steel manufacture, an overabundant amount of steel slag has become a threat to the environment in recent years. One solution to this problem is recycling and utilization of steel slag in road construction. Generally, steel slag is composed of SiO_2_, CaO, MgO, FeO_n_ and others (Al_2_O_3_, MnO, P_2_O_5_, f-CaO, f-MgO, etc.). Because of the free CaO (f-CaO), steel slag aggregate may expand during its service life and result in a performance decline on pavement, such as permanent deformation, cracking and flushing [5].

However, it was found that once the expansion is resolved by sufficient aging, steel slag can improve the performance characteristics of asphalt mixture, such as a higher dynamic modulus and tensile strength, decreased deformation and improved rutting and cracking resistance [6,7,8,9]. Research shows that aging in air for a certain time can reduce the content of f-CaO in steel slag and eliminate the effect of expansion [10]. Wu studied the utilization of steel slag aggregate in SMA mixtures and found that steel slag in SMA mixtures both enhanced the ability of resisting permanent deformation at high temperature and the resistance to low temperature cracking, compared with basalt [11]. Xie researched the recycling of basic oxygen furnace (BOF) slag in asphalt mixtures. They found that the addition of BOF could significantly improve the mechanical strength and moisture damage resistance [12,13,14]. Furthermore, SHMA shows a better skid resistance because of its high strength and irregular shape [15,16].

With the rapid development of the transportation industry, the traffic pressure on highways is climbing year by year. Driven by economic interests, overloaded trucks are appearing frequently on highways. At present, the average axle load of heavy vehicles is about 12–18 t, and the tire pressure is about 0.8–1.1 MPa [17]. When overloaded, the actual axle load of trucks could be up to 24 t and the pressure could take 100% more than its allowance, which has brought a serious burden on the pavement [18]. With the compacting, wearing and polishing of overloaded vehicle, the strength and skid resistance of pavements will decrease quickly, and it will greatly reduce their service life [19].

At present, most of the research on SHMA focuses on the performance change under a standard load, which is 0.7 MPa, while the studies on the performance degradation under heavy loading are relatively few [20,21,22,23]. It has been proven that the addition of steel slag could increase the mechanical strength of asphalt mixtures under a standard load. Therefore, it is meaningful to research whether steel slag can improve the performance of asphalt mixture under heavy loading. In this study, aggregate characteristics of basalt and steel slags are quantized and measured. The performance degradation of various SHMA under heavy loading is compared and evaluated. The rutting performance, skid resistance and aggregate grading variation are analyzed to reveal the influence of heavy loading on the performance of SHMA. The performance of BHMA under heavy loading is studied for contrast. This study proposes an explanation for the mechanism of performance degradation of SHMA under heavy loading. Those analysis results will provide significant information for decision-makers to increase available aggregates and the utilization of industrial by-products.

## 2. Materials and Experimental Program

### 2.1. Materials

The coarse aggregate in SHMA was hot-splashing steel slag produced by Sheng Xiang New Eco-friendly Materials Co. Ltd., Guangxi, China. The natural basalt aggregate was mined from Inner Mongolia, China. The chemical composition of the steel slag aggregate is listed in Table 1.

The basic properties of steel slag coarse aggregate and natural basalt coarse aggregate were tested according to GB/T 24765–2009 [24] and JTG E42–2005 [25] and shown in Table 2 and Table 3.

Due to the high number of voids in steel slag fine aggregate, the asphalt content in the mixtures would significantly increase, resulting in increasing costs and a waste of resources [7]. Therefore, the basalt fine aggregate was selected for both SHMA and BHMA.

Styrene-butadiene-styrene block copolymer (SBS) asphalt was selected as binder in asphalt pavement mixtures due to its high skid resistance, high rutting resistance and temperature stability. The basic properties of SBS asphalt are illustrated in Table 4 according to the JTG E20–2011 [26] standards.

**Figure 1:**
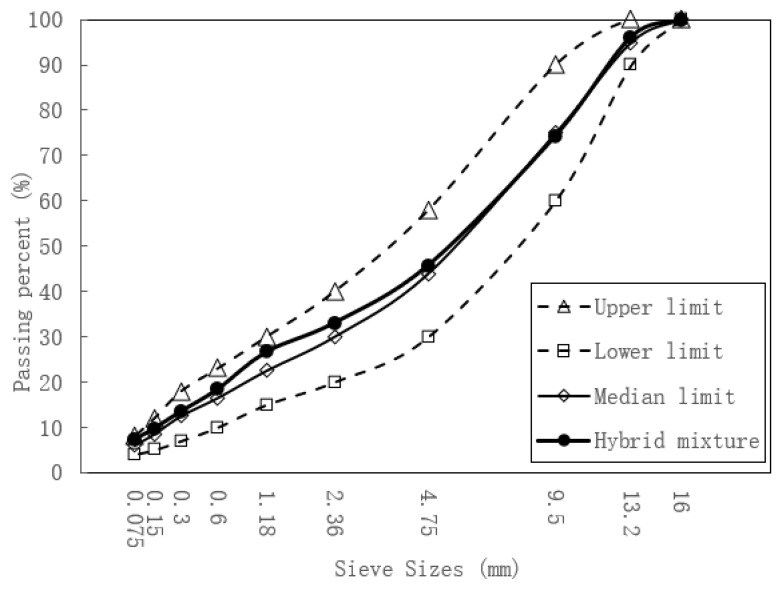
AC-13 grading curve of BHMA.

The asphalt mixture was prepared according to JTG E20-2011 [26]. The AC-13 product was applied on the upper pavement. The grading curve of BHMA is shown in Figure 1. The AC-13 BHMA gradings were 28% 10–16 mm basalt, 28% 5–10 mm, 14% 3–5 mm, 26% 0–3 mm, and 4% basalt powder filler. In order to ensure the consistency of grading, the basalt in BHMA was replaced by equal volume steel slag according to its density. The AC-13 SHMA grading reached the following values: 29% 9.5–16 mm basalt, 28% 4.75–9.5 mm, 15% 2.36–4.75 mm, 24% 0–2.36 mm, and 4% basalt powder filler.

### 2.2. Specimen Preparation

According to the content of steel slag aggregate, specimens were divided into five types—SM (steel slag mixture)-0, SM-25, SM-50, SM-75 and SM-100. SM-25 meant it contained 25% steel slag aggregate and 75% basalt aggregate. Therefore, SM-0 was pure BHMA and SM-100 was pure SHMA.

Large-sized specimens were adopted in this study to reduce the influence caused by specimen boundary [27,28]. The large-sized asphalt mixture specimens had a length of 1000 mm, width of 500 mm and height of 40 mm. Aggregate and SBS asphalt were preheated up to 165 °C and compacted by a steel roller. The asphalt content was equal to 4.4%. The final percentage of SM-0 components were 4.4% SBS bitumen, 26.8% 9.5–16 mm basalt, 26.8% 4.75–9.5 mm, 13.4% 2.36–4.75 mm, 24.8% 0–2.36 mm and 3.8% basalt powder filler. The composition of the other specimens can be calculated from the content and density of steel slag. The finished specimens were left at room temperature for 24 h to reach a stable structure and conditioned in a testing environment for at least 6 h before the test.

### 2.3. Accelerated Testing Plan

**Figure 2:**
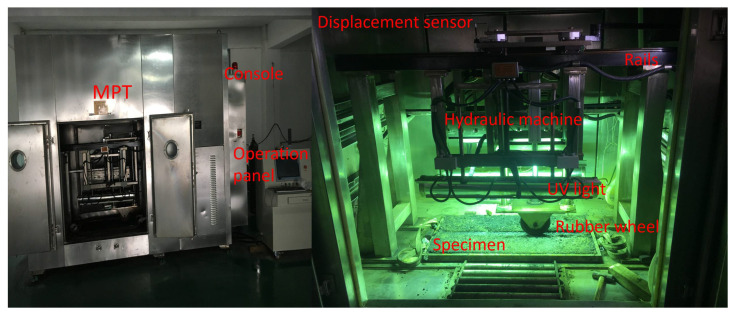
Multifunctional pavement material tester.

The tests were conducted in a multifunctional pavement material tester (MPT) developed by Wuhan University of Technology (Figure 2). Its functional part was a rubber wheel with a width of 40 mm that moved on two steel rails. The contact area was represented by an area of 1200 mm^2^. The loading cylinder applied a dynamic equilibrium pressure to the specimens, ranging from 0 to 30 MPa. The testing condition, such as temperature, UV, oxygen concentration, humidity, etc., were separately controlled by different modules. In this study, the testing conditions were set to a temperature of 60 °C, a humidity of 40% and a speed of 12 cycles/min according to relevant studies [28]. Displacement sensors recorded the real-time deformation of the specimens.

In order to simulate the effect of long-term heavy loading, accelerated testing was adopted in this study to simulate the performance degradation of SHMA under heavy loading. The time–temperature superposition and thermorheological simplicity principles were commonly used to analyze the performance of asphalt and its mixture [29]. The WLF Equation (1) is a basic empirical formula based on the free volume theory by Williams, Landel and Ferry [30].

(1) $${\mathrm{lg}\alpha }_{T}=\frac{-C_{1}(T-T_{0})}{C_{2}+(T-T_{0})}.\;$$

where C_1_ and C_2_ are constants of the corresponding materials, T is the shift temperature, T_0_ is the reference temperature, ${\mathrm{lg}\alpha }_{T}={\mathrm{lgt}}_{0}-\mathrm{lgt}$, t_0_ and t are the corresponding times of T_0_ and T.

Therefore, the reference temperature T_0_ was set to 60 °C in the MPT to accelerate the process. The shift temperature T was set to the average annual road surface temperature 54.6 °C in Wuhan, a city in central China. By substituting the constants C_1_ = 38.46 and C_2_ = 316.35 of asphalt mixture into Equation (1) [31], the corresponding relationship between the simulation time in the MPT and the real time t_0_/t = 4.655 could be obtained.

According to the investigation of traffic around the city, the volume of heavy-load trucks (axle load ≥ 1.2 MPa) was 1500 per day approximately. The vehicle model adopted a double rectangular uniformly distributed load. The coefficient of lanes was 0.5. It was assumed that the width of the tire was 215 mm, the flat ratio was 70%, and the speed was 90 km/h (25 m/s). So, the tire–ground contact area was 215 × 70% = 150.5 mm, the contact time for one point on the road was 150.5/25,000 = 0.006 s and the contact time per day was 0.006 × 2 × 1500 × 0.5 = 9 s per day. In the simulated condition, the width of rubber tire was 40 mm, the flat ratio was 75%, and the speed was 24 m/min. The contact time per hour in the MPT was calculated to be 108 s in the same way. Therefore, 1 h in the MPT could simulate 108 × 4.655/9 = 55.86 days in real condition.

### 2.4. Aggregate Analysis System

**Figure 3:**
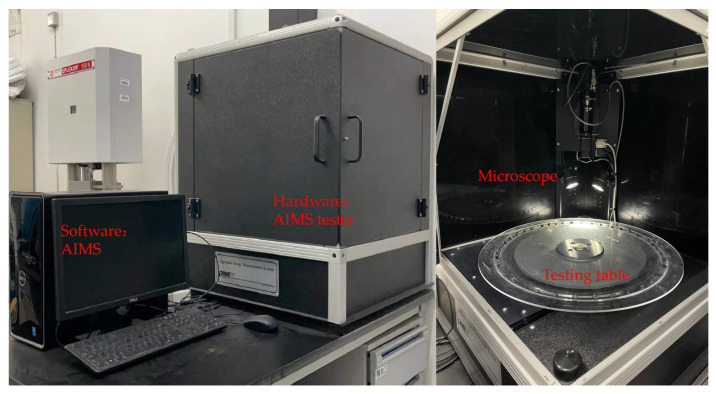
Aggregate image measurement system (AIMS) and its internal structure.

To research the different characteristics of basalt and steel slag aggregates, an aggregate image measurement system (AIMS) was used to collect data and analyze them. As shown in Figure 3, the AIMS is made up of two parts: a testing part to collect the image and a computer part to process the data. AMIS can quickly obtain and quantify aggregate shape characteristics: texture, sphericity and angularity.

Sphericity (SP) can describe the overall three-dimensional shape of a particle and determine whether it tends to be a sphere. Sphericity has a relative scale of 0 to 1 with a perfect sphere approaching a value of 1. SP can be calculated with Equation (2).

(2) $$\mathrm{SP}=\sqrt[{3}]{\frac{d_{S}d_{I}}{d_{L}^{2}}}.\;$$

where: d_S_ = particle shortest dimension; d_I_ = particle intermediate dimension; d_L_ = particle longest dimension.

Gradient angularity (GA) describes whether particle boundaries are continuous and smooth. Higher gradient values indicate a more angular shape. Gradient angularity has a relative scale of 0 to 10,000 with an ideal circle having a value of 0. The GA is analyzed by quantifying the change in the gradient on a particle boundary and is related to the sharpness of the corners in two-dimensional images of aggregate particles. The gradient method starts by calculating the inclination of gradient vectors on particle boundary points from the x-axis (horizontal axis in an image). GA can be calculated by Equation (3).

(3) $$\mathrm{GA}=\frac{1}{\frac{n}{3}-1}\overset{n-3}{\underset{i=1}{\sum }}\left|\theta_{i}-\theta_{i+3}\right|$$

where: θ = angle of orientation of the edge points; n = the total number of points; subscript i denoting the ith point on the edge of the particle.

Texture (TX) describes the relative smoothness or roughness of aggregate particles’ surfaces. AIMS uses the wavelet method to quantify texture. The wavelet analysis gives the texture details in the horizontal, vertical, and diagonal directions in three separate images. The texture index at a given decomposition level is the arithmetic mean of the squared values of the wavelet coefficients for all three directions. Texture has a relative scale of 0 to 1000 with a smooth polished surface having a value of 0. The texture index is expressed mathematically as Equation (4).

(4) $$\mathrm{TX}=\frac{1}{3N}\sum_{i=1}^{3}\sum_{j=1}^{N}{\left(D_{i,j}\left(x,y\right)\right)}^{2}$$

where: D = decomposition function; n = decomposition level; N = total number of coefficients in an image; i = 1, 2 or 3 for detailed images; j = wavelet index; x,y = location of the coefficients in the transformed domain.

### 2.5. Testing Projects and Methods

A survey report about Chinese highway transportation showed that the pressure applied by heavy trucks on the pavement was generally between 1.2 MPa and 1.7 MPa. Therefore, a value of 1.4 MPa was selected to simulate the overload condition [32]. SHMA specimens would be ground for 12–108 h under set point to observe the degradation. Each specimen would undergo 8640–77,760 times of wheel rolling. According to the accelerating test above, those tests could simulate 2–16 years of service life in real condition.

Permanent deformation is connected with the mechanical strength of an asphalt pavement. The test can evaluate the stability and durability of the mixture. Texture depth (TD) and British pendulum number (BPN) can reflect the texture and skid resistance of the pavement indirectly [33]. The test of TD and BPN can compendiously describe the micro and macro texture and friction coefficient, respectively. Therefore, permanent deformation, TD and BPN were selected as three indexes to measure the degradation of SHMA under heavy loading. The specimens were divided and dissolved by methylbenzene to separate aggregate from asphalt binder. The separate aggregate was sieved to analyze the variation of steel slag aggregate after heavy loading.

**Figure 4:**
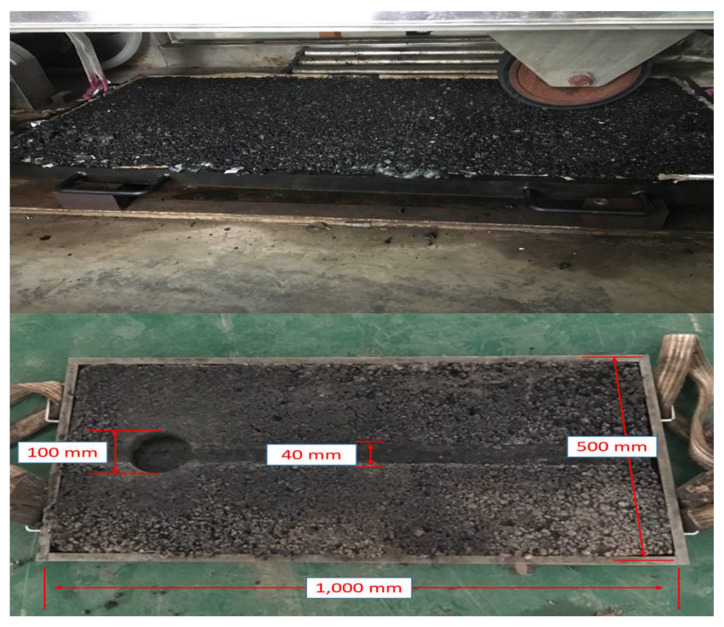
Specimens in and after the test.

Figure 4 presents the prepared specimens in and after the fatigue test. The ground parts of the specimens were extracted to study the degradation under heavy loading.

## 3. Experimental Results and Discussion

### 3.1. Aggregate Analysis Results

Coarse aggregates of sizes 4.75–9.5 mm and 9.5–16 mm were selected as a typical example to research the particle characteristics using the AIMS. The aggregate analysis results are shown in Table 5.

As shown in Table 4, for the same aggregate, SP and GA decreased with the decline of aggregate particle size, while TX increased. By comparing the particle characteristics of basalt and steel slag, it could be found that steel slag aggregate had a lower SP, higher GA and TX. Analysis results illustrated there was not much difference between basalt and steel slag in sphericity, but steel slag was significantly higher than basalt in angularity and texture. Steel slag aggregate had the particle characteristics of low sphericity, high angularity and rich surface texture.

**Figure 5:**
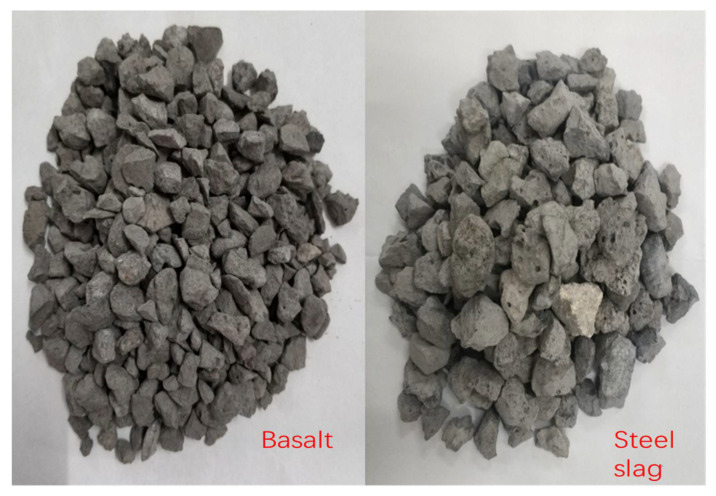
Basalt and steel slag aggregate.

Figure 5 showed the typical characteristics of basalt aggregate and steel slag aggregate. It can also be observed in the figure that basalt aggregate had a smoother surface and fewer edges. Conversely, there were abundant pores and edges on the surface of the steel slag aggregate.

### 3.2. Results from Fatigue Test

**Figure 6:**
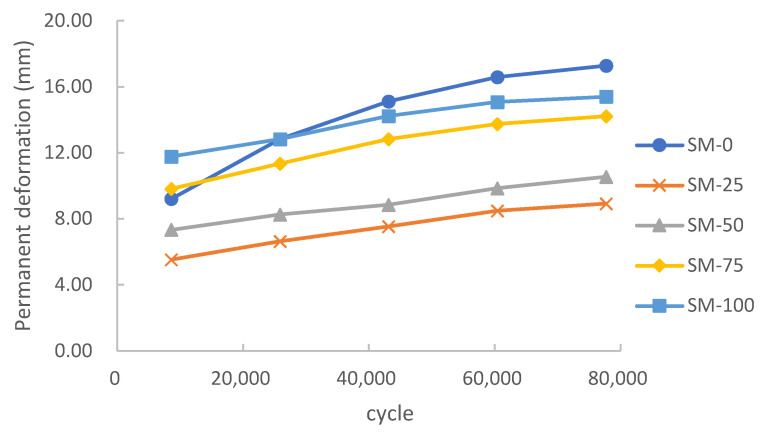
Effect of steel slag content on permanent deformation.

Figure 6 shows the effects on the development of rutting of various asphalt pavements under a pressure of 1.4 MPa. Each series was constituted by five periods of cumulative cycles.

Normally, the rutting development of asphalt pavement can be divided into three stages: initial stage, steady stage and destruction stage [34]. In the first stage, the permanent deformation increases gradually, then it goes into the second stage, a period of stability. The rutting grows slowly at a steady rate during this time. Finally, the rutting and its growth rate continue to increase until its failure.

As shown in Figure 6, the permanent deformation curve of four specimens with steel slag were in the steady stage during the test. This illustrated that they passed through their initial stage before the first date collection (8640 cycles). Comparatively, the only specimens without steel slag added, SM-0, was still in its initial stage at the beginning of the test. According to the trend of the SM-0 curve, it would have ended its initial stage at around 40,000 cycles of wheel rolling.

By comparing the initial (8640 cycles) and final (77,760 cycles) deformation values of five series, it could be found that the initial value of most series was about 60–70% of the final value, except for the SM-0 series without the addition of steel slag, whose initial value was only 53% of the final value.

The wheel tracking slope (WTS) could be used to describe the increase in permanent deformation [35]. The WTS was calculated by Equation (5).

(5) $$\mathrm{WTS}=\frac{h_{\mathrm{cycle}1}-h_{\mathrm{cycle}2}}{\mathrm{cycle}1-\mathrm{cycle}2}$$

where h_cycle_ is the depth of deformation in the cycle.

According to the calculation, the WTS of the four series with steel slag was about 0.5–0.6 mm/(104 cycles), which was much less than the WTS of 1.17 mm/(104 cycles) in the SM-0 series. This illustrates that the addition of steel slag could significantly decrease the evolution of permanent deformation on the pavement.

According to JTJ073.2–2001 [36], the allowance of deformation is no higher than 15 mm on highways. Once the deformation exceeds 15 mm, the pavement is at risk of failure and needs major repair. Experimental results showed that the asphalt mixture with a part of steel slag (SM-25, SM-50, SM-75) would not exceed the limit under heavy loading in long-term service time. However, the mixture without steel slag (SM-0) and the mixture with pure steel slag (SM-100) were unable to be in service under heavy loading for long. This indicated that partial replacement of steel slag improved the performance, but all replacements resulted in a performance decline.

The above results showed that the addition of steel slag could significantly accelerate the process of the initial stage and reduce the final permanent deformation of the asphalt pavement, enhancing its mechanical properties, especially at low amounts of steel slag addition. According to the test results, the optimum addition amount of steel slag was 25%, which could reduce the permanent deformation by 48.2%. Due to its higher strength and angularity and richer surface texture, steel slag could bond better with asphalt binder at low amounts of steel slag addition, which provided stronger mechanical properties. However, with the increase of steel slag content, the voids in the steel slag absorbed asphalt in abundance. This decreased the amount of asphalt that acted as binder, resulting in a strength decline of the asphalt mixture.

**Figure 7:**
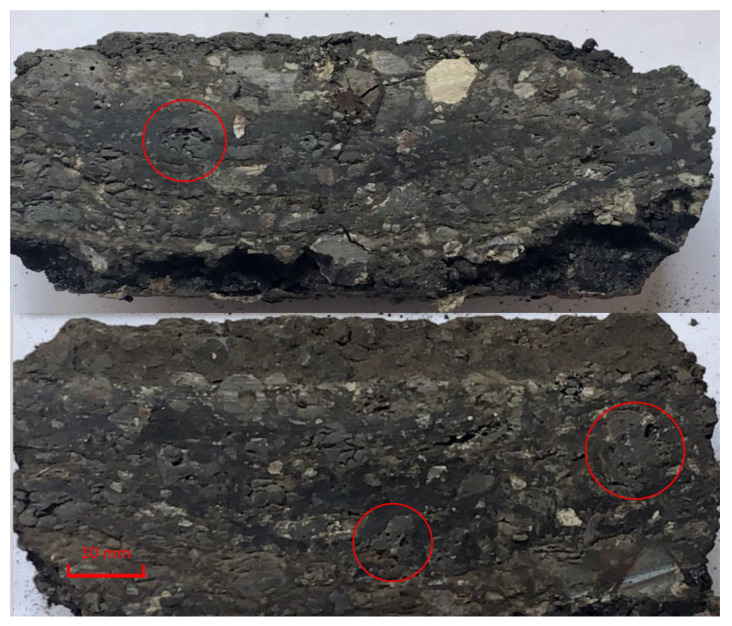
Cross-section view of SHMA.

Figure 7 shows two cross-section views of SHMA after a wheel-rolling test. The angularity and voids of the steel slag are presented in the figure.

### 3.3. Skid Resistance Results

**Figure 8:**
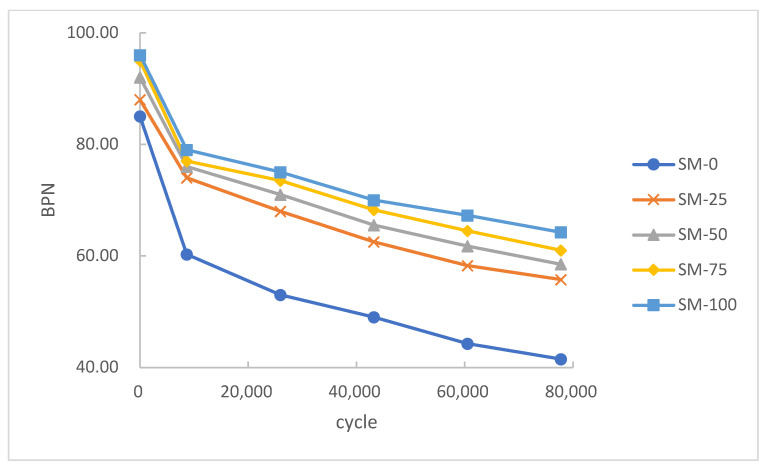
Effect of steel slag content on BPN.

Figure 8 shows the variation in BPN of the asphalt mixture with different content of steel slag aggregate. Generally, a large BPN means that the pavement can provide higher friction, which means a better skid resistance.

As presented in Figure 8, at the initial stage, BPN decreased rapidly and stabilized gradually. In all five series, the BPN of SM-0 was much lower than in the other series. This result suggested that the addition of steel slag could significantly improve the skid resistance of the asphalt mixture. Moreover, the addition of steel slag could also contribute to reducing skid resistance loss. Experimental results showed that the final BPN of SM-0 decreased by 51.2%. However, the final BPN of the other series with steel slag decreased by 33.3–36.6% to a varying degree.

It was found that the five series had a similar initial BPN. After a period of wheel grinding, the asphalt mixture on the surface was compacted to expose the edges of the inner aggregate. After that, the aggregate corners and edges without mixture protection were gradually destroyed by the wheel, which resulted in the rapid decline of skid resistance. Due to its higher strength and angularity, steel slag could retain more edges and corners after wheel grinding under heavy loading. Therefore, the higher content of steel slag could provide better skid resistance.

According to JTJ073.2–2001 [36], the BPN on highway should be higher than 45. Obviously, SM-0, the specimen without addition of steel slag, could not meet the requirement of long-term service under heavy loading. Meanwhile, other asphalt mixtures with steel slag can satisfy this requirement.

**Figure 9:**
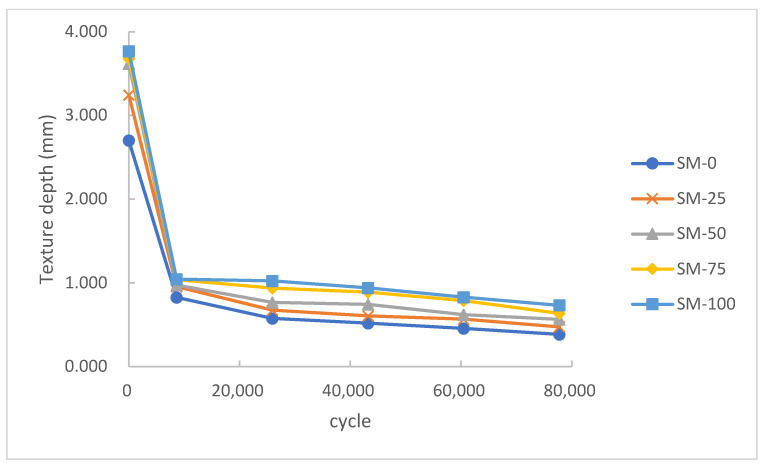
Effect of steel slag content on texture depth.

The TD showed a similar variation to BPN according to Figure 9. The asphalt mixture was compacted to expose the corners and edges of aggregates on the surface at the beginning. The destruction of the initial structure resulted in the rapid decline of the TD. Then, the edges and corners on the aggregates were ground off and the aggregates were gradually crushed into pieces by heavy loading, leading to a further decline of the TD in the later stage. In this process, the abundant pores in the steel slag could enrich the surface texture of the asphalt mixture. However, this improvement was not noticeable under long-term heavy loading. Therefore, the TD increased slightly with the increase of steel slag content.

The skid resistance degradation mechanism of SHMA can be summarized according to the experimental results. The steel slag aggregate on the surface of the mixture was ground and the edges were worn off due to its particle characteristics under heavy loading. The polished aggregate led to a decline in skid resistance. However, because of the better angularity and texture of steel slag, the skid resistance of SHMA was still superior to BHMA after heavy loading service.

According to some relevant studies, the TD of an asphalt mixture mainly depends on its type and curve of aggregate grading [37]. Compared with the grading type, the coarse aggregate type had little effect on skid resistance. Therefore, the aggregate grading type should be given priority to improve the skid resistance. However, replacing coarse aggregate with steel slag could be a good supplement to the skid resistance of the asphalt mixture.

According to JTG D50-2006 [38], the allowance for TD on highways should be higher than 0.55 mm. Likewise, asphalt mixtures without steel slag were not able to serve in the long term under heavy loading. The mixtures with steel slag could accomplish this task well.

### 3.4. Sieving Results

Organic solvents were used to dissolve the asphalt in the samples to obtain the variation of aggregate grades after heavy loading test. Table 6 shows the sieving results of asphalt mixtures that underwent 108 h of heavy loading testing. In each series, 0# indicates the initial grade.

Due to the randomness of the sampling, these sieving results could not accurately describe the real grading, but they could reflect the changing trend of the grading under heavy loading. As shown in Table 6, the fine aggregates (0–2.36 mm) changed insignificantly after the test. Considering the loss of powdery aggregates in the separation process, it could be assumed that the fine aggregates had no loss or increase in the whole process.

As shown in Table 6, the content of the 9.5–16 mm, 4.75–9.5 mm, 2.36–4.75 mm aggregates in the mixtures changed significantly. The content of the 9.5–16 mm part decreased while the 4.75–9.5 mm and 2.36–4.75 mm parts increased. These results illustrate that the large-particle aggregates in the asphalt mixtures were crushed into pieces under heavy loading. It could also be observed in the cross section of Figure 7.

The 9.5–16 mm aggregate was pressed by heavy loading and broke into smaller pieces. The 4.75–9.5 mm aggregate was crushed by heavy loading and reduced, on the one hand; On the other hand, it received pieces from the crushed 4.75–9.5 mm aggregate and increased. As the result, the content of the 4.75–9.5 mm aggregate increased as well as the 2.36–4.75 mm aggregate. It could be found that the increments of 4.75–9.5 mm and 2.36–4.75 mm aggregates were about half of the loss of the 9.5–16 mm aggregate. As a result, it could be assumed that half of the crushed 9.5–16 mm aggregate became 4.75–9.5 mm aggregate and the other half became 2.36–4.75 mm aggregate. The main change in asphalt mixture was the crushing of the 9.5–16 mm aggregate.

**Figure 10:**
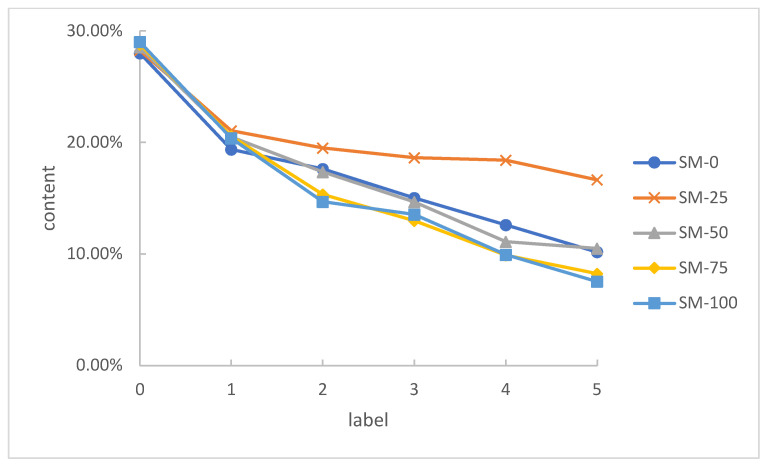
Variations in 9.5–16 mm aggregate content.

The variation in 9.5–16 mm aggregate is demonstrated in Figure 10. It could be observed that the final content of 9.5–16 mm aggregate after heavy loading decreased with the increase of the steel slag content. Although steel slag had a higher strength, its irregular shape caused stress concentration and broke more easily. Therefore, steel slag was more likely to be crushed under heavy loading than basalt aggregate.

SM-25 had the highest final content of 9.5–16 mm aggregate. Moreover, SM-25 had the highest deformation resistance according to the permanent deformation results. In order to verify whether there is a correlation between permanent deformation and the content of 9.5–16 mm aggregate, a Pearson correlation coefficient (PCC) test was conducted by SPSS (Statistical Product and Service Solutions). The PCC test is a measure of the linear correlation between two variables, X and Y. The PCC has a value between 1 and −1, where 1 is total positive correlation, 0 is no correlation, and −1 is total negative correlation. The PCC can be computed by Equation (6).

(6) $$r_{\mathrm{xy}}=\frac{\sum_{i=1}^{n}\left(x_{i}-\bar{x}\right)\left(y_{i}-\bar{y}\right)}{\sqrt{\sum_{i=1}^{n}{\left(x_{i}-\overset{ˉ}{x}\right)}^{2}}\sqrt{\sum_{i=1}^{n}{\left(y_{i}-\overset{ˉ}{y}\right)}^{2}}}$$

where $r_{\mathrm{xy}}$ is the PCC; n is the sample size; xi, yi are the individual samples; $\overset{ˉ}{x}=\frac{1}{n}\sum_{i=1}^{n}x_{i}$, analogously for $\;\bar{y}$.

The contents of 9.5–16 mm, 4.75–9.5 mm and 2.36–4.75 mm aggregates were taken as variables to conduct the PCC test with permanent deformation. The analysis results are shown in Table 7.

It can be seen that the content of 9.5–16 mm aggregate had the highest PCC with permanent deformation, and it could pass a significance test at the 0.01 level. It illustrated that there was a fairly strong correlation between the content of 9.5–16 mm aggregate and the permanent deformation of asphalt mixture.

The large aggregates in asphalt mixtures with a high content of steel slag were easier to crush under heavy loading. Therefore, the asphalt with a high content of steel slag did not have enough large aggregates to support its strength after heavy loading service. That could be another important reason why the deformation resistance of asphalt mixtures with a high content of steel slag was inferior to those asphalt mixtures with a low content of steel slag.

The mechanism of degradation of SHMA can be summarized based on the above results. Large steel slag aggregates were crushed under heavy loading due to their particle characteristics. The broken aggregates moved in the mixture and formed a new stable skeleton structure, which led to the decline of deformation resistance.

## 4. Conclusions

Large-sized specimen wheel tracking testing on AC-13 asphalt mixtures with various contents of steel slag was carried out to study the performance degradation under heavy loading conditions. The following results were obtained:

(1) Steel slag aggregates have high strength, abundant angularity and rich surface texture characteristics. The degradation of asphalt mixtures under heavy loading can be effectively reduced after the addition of steel slag.
(2) The addition of steel slag aggregate to the asphalt mixture can reinforce the resistance to deformation and skid under heavy loading. Permanent deformation on SHMA can be decreased by 48.2% with a low content of 25% steel slag. A high steel slag content reduces the asphalt that acts as a binder, resulting in the loss in strength of the asphalt mixture. The abrasion and polishing of steel slag aggregate on the surface was a main cause for the decline of skid resistance under heavy loading. The performance of SHMA can still meet the pavement technical requirement after long-term heavy loading service.
(3) The sieving results indicate that the main change that occurs in the asphalt mixture under heavy loading service is the crushing of the 9.5–16 mm aggregate. The fracture of large particles forms a new aggregate grading and changes the properties of the asphalt mixture. The content of 9.5–16 mm aggregate has a great influence on permanent deformation. Steel slag is more likely to be broken into smaller particles under heavy loading, which is also an important reason for the lower resistance to deformation of the asphalt mixture with a high content of steel slag.
(4) The mechanism of deformation resistance degradation of SHMA under heavy loading can be summarized as follows: the large aggregate was crushed and formed a new aggregate skeleton structure.

## Figures and Tables

**Table 1:** Main chemical composition of the steel slag aggregate.

Chemical Composition	SiO_2_	CaO	MgO	FeO_n_	Al_2_O_3_	MnO	P_2_O_5_
Steel slag	19.40	41.60	5.36	22.40	3.12	1.57	1.29

**Table 2:** Basic properties of steel slag coarse aggregate.

Test Items		Units	Results	Requirements	Specification
Apparent specific gravity	9.5–16 mm		3.386	≥2.9	T0304–2005
	4.75–9.5 mm		3.426		
	2.36–4.75 mm		3.465		
Water absorption	9.5–16 mm	%	1.7	≤3.0	
	4.75–9.5 mm		2.4		
	2.36–4.75 mm		2.9		
Crushed stone value		%	18.9	≤22	T0316–2005
Los Angeles abrasion		%	20.4	≤22	T0317–2005
Adhesion level			5	≥4	T0616–2000
Polished stone value		PSV	61.5	≥45	T0312–2005

**Table 3:** Basic properties of basalt coarse aggregate.

Test Items		Units	Results	Requirements	Specification
Apparent specific gravity	9.5–16 mm		2.974	≥2.6	T0304–2005
	4.75–9.5 mm		2.995		
	2.36–4.75 mm		2.908		
Water absorption	9.5–16 mm	%	0.4	≤2.0	
	4.75–9.5 mm		0.7		
	2.36–4.75 mm		0.7		
Crushed stone value		%	19.2	≤22	T0316–2005
Los Angeles abrasion		%	22.7	≤22	T0317–2005
Adhesion level			4	≥4	T0616–2000
Polished stone value		PSV	55.2	≥42	T0312–2005

**Table 4:** Basic properties of SBS asphalt binder.

Test Items		Results	Requirements	Specification
Penetration 25 °C, 100 g, 5 s (0.1 mm)		76.2	60~80	T0604–2011
Softening point (°C)		63.6	≥60	T0606–2011
Ductility 5 cm/min, 5 °C (cm)		51.2	≥30	T0605–2011
Density 15 °C (g/cm^3^)		1.031	/	T0625–2011
Solubility (TCE) (%)		99.8	≥99	T0607–2011
Aging 163 °C, 5 h	Mass change (%)	0.4	±1.0	T0610–2011
	Penetration ratio 25 °C (%)	83.3	≥60	T0604–2011
	Residual ductility 5 °C (cm)	32.3	≥20	T0605–2011F

**Table 5:** Aggregate analysis results.

Aggregate		SP	GA	TX
Basalt	9.5–16 mm	0.735	2965	356
	4.75–9.5 mm	0.673	2846	421
Steel slag	9.5–16 mm	0.704	3264	718
	4.75–9.5 mm	0.636	3151	771

**Table 6:** Sieving results of ground asphalt mixtures.

Specimen	Particle Size	Label					
		0	1	2	3	4	5
SM-0	9.5–16 mm	28.00%	19.38%	17.62%	15.01%	12.61%	10.17%
	4.75–9.5 mm	28.00%	33.34%	33.65%	34.26%	36.22%	36.40%
	2.36–4.75 mm	14.00%	19.51%	19.85%	22.87%	23.02%	23.37%
	0–2.36 mm	30.00%	27.77%	28.89%	27.86%	28.16%	30.06%
SM-25	9.5–16 mm	28.26%	21.04%	19.50%	18.63%	18.40%	16.65%
	4.75–9.5 mm	28.11%	32.57%	33.25%	32.96%	32.67%	32.68%
	2.36–4.75 mm	14.32%	19.61%	21.99%	21.49%	21.05%	23.70%
	0–2.36 mm	29.37%	26.78%	25.26%	26.92%	27.88%	26.97%
SM-50	9.5–16 mm	28.53%	20.49%	17.36%	14.66%	11.10%	10.50%
	4.75–9.5 mm	28.22%	31.01%	36.50%	37.20%	39.92%	38.54%
	2.36–4.75 mm	14.62%	21.47%	21.08%	22.68%	23.51%	24.74%
	0–2.36 mm	28.63%	27.03%	25.06%	25.46%	25.47%	26.22%
SM-75	9.5–16 mm	28.79%	20.56%	15.34%	12.98%	9.88%	8.23%
	4.75–9.5 mm	28.33%	31.25%	35.64%	36.78%	38.65%	39.42%
	2.36–4.75 mm	14.92%	21.23%	22.65%	23.51%	24.32%	25.41%
	0–2.36 mm	27.96%	26.96%	26.37%	26.73%	27.96%	26.94%
SM-100	9.5–16 mm	29.00%	20.37%	14.68%	13.55%	9.93%	7.53%
	4.75–9.5 mm	29.00%	31.26%	34.84%	33.47%	38.19%	39.84%
	2.36–4.75 mm	15.00%	20.78%	21.43%	23.88%	24.55%	26.34%
	0–2.36 mm	27.00%	27.59%	29.04%	29.10%	27.33%	26.29%

**Table 7:** PCC test results.

	9.5–16 mm	4.75–9.5 mm	2.36–4.75 mm
PCC	−0.727	0.475	0.569
Sig.	0.000039	0.16	0.003
